# Epigenetics Analysis and Integrated Analysis of Multiomics Data, Including Epigenetic Data, Using Artificial Intelligence in the Era of Precision Medicine

**DOI:** 10.3390/biom10010062

**Published:** 2019-12-30

**Authors:** Ryuji Hamamoto, Masaaki Komatsu, Ken Takasawa, Ken Asada, Syuzo Kaneko

**Affiliations:** 1 Division of Molecular Modification and Cancer Biology, National Cancer Center Research Institute, 5-1-1 Tsukiji, Chuo-ku, Tokyo 104-0045, Japan; maskomat@ncc.go.jp (M.K.); ktakazaw@ncc.go.jp (K.T.); ken.asada@riken.jp (K.A.); sykaneko@ncc.go.jp (S.K.); 2 Cancer Translational Research Team, RIKEN Center for Advanced Intelligence Project, 1-4-1 Nihonbashi, Chuo-ku, Tokyo 103-0027, Japan

**Keywords:** epigenetics, precision medicine, DNA methylation, histone modifications, machine learning, deep learning

## Abstract

To clarify the mechanisms of diseases, such as cancer, studies analyzing genetic mutations have been actively conducted for a long time, and a large number of achievements have already been reported. Indeed, genomic medicine is considered the core discipline of precision medicine, and currently, the clinical application of cutting-edge genomic medicine aimed at improving the prevention, diagnosis and treatment of a wide range of diseases is promoted. However, although the Human Genome Project was completed in 2003 and large-scale genetic analyses have since been accomplished worldwide with the development of next-generation sequencing (NGS), explaining the mechanism of disease onset only using genetic variation has been recognized as difficult. Meanwhile, the importance of epigenetics, which describes inheritance by mechanisms other than the genomic DNA sequence, has recently attracted attention, and, in particular, many studies have reported the involvement of epigenetic deregulation in human cancer. So far, given that genetic and epigenetic studies tend to be accomplished independently, physiological relationships between genetics and epigenetics in diseases remain almost unknown. Since this situation may be a disadvantage to developing precision medicine, the integrated understanding of genetic variation and epigenetic deregulation appears to be now critical. Importantly, the current progress of artificial intelligence (AI) technologies, such as machine learning and deep learning, is remarkable and enables multimodal analyses of big omics data. In this regard, it is important to develop a platform that can conduct multimodal analysis of medical big data using AI as this may accelerate the realization of precision medicine. In this review, we discuss the importance of genome-wide epigenetic and multiomics analyses using AI in the era of precision medicine.

## 1. Introduction

Barack Obama, the 44th president of the United States, stated his intention to fund an amount of $215 million to the “Precision Medicine Initiative” in his 2015 State of the Union Address [1]. Since then, precision medicine has frequently been used as a term that contains concepts of personalized medicine worldwide. Generally, precision medicine refers to a medical model that proposes the customization of healthcare with medical decisions, treatments, practices or products tailored to individual patients. In this model, diagnostic testing is often employed for selecting appropriate and optimal therapies based on the context of a patient’s genetic content or other molecular or cellular analyses [2]. To date, most precision medicine interventions consist of genetic profiling, including the detection of predictive biomarkers [3]. It has been repeatedly reported that this may identify patients at risk for a specific disease or a severe variant of a disease and allow for preventive interventions to reduce the burden of disease and improve quality of life. However, it has also been reported that only a small number of patients benefit from current precision medicine, and it is of no benefit for most tumor patients [4,5]. In addition, it has been stated that the MD Anderson Cancer Center found that the gene sequencing of 2600 people only benefited 6.4% of them through the use of targeted drugs. According to the data about matching plans of the National Cancer Institute, only 2% people can benefit from targeted drugs [4,6]. These results indicate that we definitely need to explore the possibility that more patients can benefit from precision medicine. To extend precision medicine, not only genomic data but also other omics data, such as epigenetic and proteomics data, should be involved, and integrated analyses of different types of omics data are considered to be of paramount importance. In this review article, we highlight the current knowledge of the importance of epigenetic data in precision medicine by describing, in particular, the integrated analysis of multiomics data, including epigenetic data, using machine learning and deep learning technologies.

## 2. Characteristics of Epigenetics and Technologies for Epigenetics Analysis

### 2.1. General Characteristics of Epigenetics

**Figure 1:**
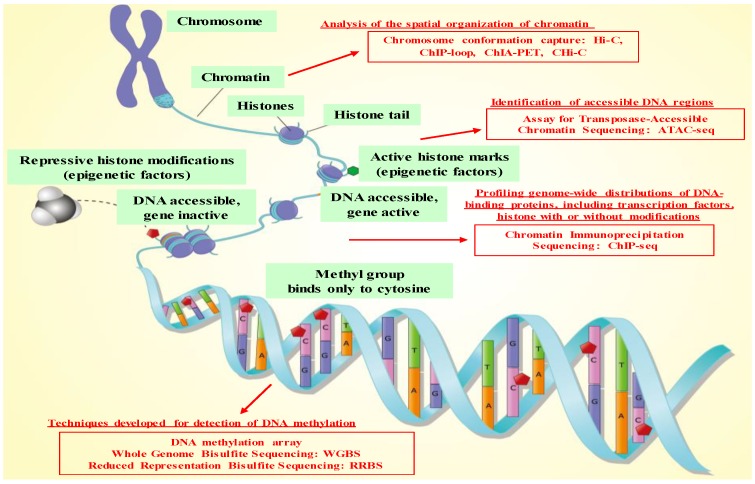
The summarized figure of epigenetic regulations and technologies for epigenetics analysis. Image credit: Shutterstock.com/ellepigrafica.

In principle, epi-genetics is the study of heritable phenotype changes without altering the DNA sequence [7]. The Greek prefix epi- (ἐπι- “above”) in epi-genetics implies features that are “on top of” or “in addition to” the traditional genetic basis for inheritance [8]. Over the last decade, epigenetic regulators have been implicated as key factors in many pathways relevant to cancer development and progression, including cell cycle regulation, invasiveness, signaling pathways, chemo-resistance and immune evasion [9,10,11,12,13,14,15,16,17,18,19,20,21,22,23,24,25,26,27,28,29,30,31,32,33,34,35,36,37,38]. The three basic systems of epigenetic regulation are DNA methylation of gene regulatory regions, histone protein modifications, such as methylation, acetylation, phosphorylation and sumoylation and non-coding RNAs [15,20,21]. With regard to the technologies for epigenetics analysis, a number of methods have already been developed, and this field has made steady progress in technological innovation (Figure 1 and Table 1). Below, we highlight technologies for epigenetics analysis including historical context.

### 2.2. Technologies for Epigenetics Analysis before the NGS Era

In the 1980s, the basic principle of chromatin immunoprecipitation (ChIP) was established; for instance, Gilmour and Lis demonstrated that proteins were cross-linked to DNA in intact cells, and the protein-DNA adducts were isolated by immunoprecipitation with antiserum against the protein [39]. On the basis of this principle, several kinds of applied technologies were reported so far, indicating that this methodology greatly contributes to the progress of epigenetics. In the 1990s, as understandings of the physiological and biological importance of the DNA methylation were deepened, assay methods to analyze DNA methylation status were actively developed. Importantly, treatment of DNA with bisulfite converts cytosine residues to uracil, but leaves 5-methylcytosine residues unaffected. Hence, DNA that has been treated with bisulfite retains only methylated cytosines. On the basis of this molecular mechanism, Frommer et al. reported a genomic sequencing method that provides positive identification of 5-methylcytosine residues and yields strand-specific sequences of individual molecules in genomic DNA [41], which formed the basis of subsequent development of DNA methylation assays. At the end of the 20th century, DNA microarray-based methods were also used for epigenetics analysis. In 1999, a DNA-array-based method, called differential methylation hybridization (DMH), was developed to identify hypermethylated sequences in tumor cells by simultaneously screening many CpG islands (CGIs) [45]; this technology could explore further the underlying mechanisms of DNA methylation. Likewise, a first ChIP-on-chip experiment, a technology that combines chromatin immunoprecipitation (ChIP) with DNA microarray (chip), was performed in 1999 to analyze the distribution of cohesin along budding yeast chromosome III [47]. Using tiled arrays, ChIP-on-chip allows for high resolution of genome-wide maps, which can determine the binding sites of many DNA-binding proteins like transcription factors and also chromatin modifications.

As for biochemical analysis of epigenetic regulators, such as histone acetyltransferases, histone methyltransferases and histone demethylases, various procedures were developed in the late 20th and early 21st centuries [43,49,51,76]. A series of biochemical analyses particularly unveiled the biological significance of epigenetics so far.

### 2.3. Technologies for Epigenetics Analysis in the NGS Era and Genome-Wide Epigenetics Analysis

In 2005, new sequencing techniques began to emerge that permitted an unbiased means to examine billions of templates of DNA and RNA. Although now almost fifteen years old, the term “next-generation sequencing (NGS)” remains the popular way to describe very-high-throughput sequencing methods that allow millions to trillions of observations to be made in parallel during a single instrument run [77]. Importantly, progress of NGS technologies produced several methods of genome-wide epigenetics analysis. Reduced representation bisulfite sequencing (RRBS) is an efficient and high-throughput technique to analyze the genome-wide methylation profiles; it combines restriction enzymes and bisulfite sequencing to enrich for areas of the genome with a high CpG content. Given the high cost and depth of sequencing to analyze methylation status in the whole genome, the RRBS technique was developed in 2005 to reduce the amount of nucleotides required for sequence to 1% of the genome [53]. Moreover, in 2009, the first human genome-wide single-base-resolution DNA methylation map was established by Whole Genome Bisulfite Sequencing (WGBS) [59], which showed the utilization of this technique to investigate the relationship between DNA methylation loci and human phenotypes in both basic and clinical research [78,79].

In terms of ChIP analysis, a new method called ChIP-sequencing (ChIP-seq), which combines chromatin immunoprecipitation with massively parallel DNA sequencing (NGS), was developed in 2007 [57]. This technique enabled genome-wide analysis of histone modifications and transcription factor binding, which could contribute to the investigation of the relationship between histone modification status or transcription factor binding status and human phenotypes in both basic and clinical research [25,26,80]. Subsequently, in 2013, a new technology called ATAC-seq (assay for transposase-accessible chromatin using sequencing) was developed [65]; ATAC-seq could identify accessible (open) chromatin regions with hyperactive mutant Tn5 Transposase that inserts sequencing adaptors into open regions of the genome [81]. This method has been applied to defining the genome-wide chromatin accessibility landscape in human cancers [82], and computational footprinting methods can be performed on ATAC-seq to identify cell specific binding sites of transcription factors and their cell specific activity [83].

Furthermore, the concept of chromatin contact mapping, or determining the three-dimensional structure conformation and interactions of chromatin domains, recently attracts a lot of attention because chromosome conformation capture methods (3C-based methods) have advanced rapidly. For example, ChIP-loop is a technique that 3C-based methods and ChIP-seq are combined, which detects interactions between two loci of interest mediated by a protein of interest [55]. In addition, the Hi-C technique, a comprehensive technique to capture the conformation of genomes, is the first of the 3C derivative technologies to be truly genome-wide [61]. Subsequently, another new technology called ChIP-PET, which combines Hi-C with ChIP-seq, was developed to detect all interactions medicated by a protein of interest [55,63]. More recently, a new technique called Capture Hi-C (CHi-C) was developed (Table 1). The CHi-C method allow the simultaneous and higher resolution mapping of chromatin interactions for large subsets of the genome, such as all promoters or DNase hypersensitive sites.

In the NGS era, although genome-wide epigenetics analyses are enabled, the amount of data we need to analyze is rapidly increasing. Besides, given that multimodal analysis to integrate epigenetic data and other omics data like genomics data has recently been considered important, we recognize the importance of artificial intelligence (AI) utilization to analyze the epigenetic data efficiently and effectively.

## 3. Development of Artificial Intelligence (AI)

### 3.1. Machine Learning Techniques and Evolution of AI Technologies

**Figure 2:**
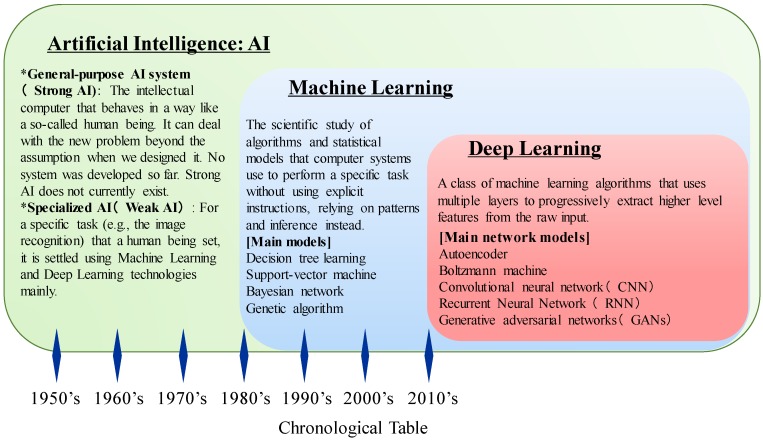
The summarized figure of artificial intelligence development.

Machine learning is a sub-set of AI technologies where computer algorisms are used to autonomously learn from data and information (Figure 2). Historically, the learning behaviors of neurons have been researched for a long time to reveal the mechanism of human cognition. One of the most famous theory is the Hebbian Learning Rule proposed by Donald Olding Hebb [84]. On the basis of the Hebbian Learning Rule in the study of artificial neural networks, we can obtain powerful models of neural computation that might be close to the function of structures found in neural systems of many diverse species [85,86]. In 1958, Frank Rosenblatt developed the perceptron, which became the first model that could learn the weights defining the categories given examples of inputs from each category [87]. In the 1980s, Kunihiko Fukushima proposed the neocognitron, which is a hierarchical, multilayered artificial neural network [88]. This neural network has been used for handwritten character recognition and other pattern recognition tasks; importantly, it served as the inspiration for convolutional neural networks [89]. In 1986, David Rumelhart, Geoff Hinton and Ronald J. Williams demonstrated the process of backpropagation, which is a method used in artificial neural networks to calculate the error contribution of each neuron after a batch of data (in image recognition, multiple images) is processed [90]. This method is a special case of an older and more general technique called automatic differentiation. With regard to the learning, it is generally used by the gradient descent optimization algorithm to tune the weight of neurons by calculating the gradient of the loss of function. Then, in 1992, Christopher Watkins developed Q-learning [91], exceedingly improving the practicality and feasibility of reinforcement learning, which is a paradigm that aims to model the trial-and-error learning process that is needed in many problem situations where explicit instructive signals are not available [92]. Additionally, Corinna Cortes and Vladimir Vapnik developed the support vector machine (SVM) machine learning algorithm, which is a model with associated learning algorithms that analyzes data used for classification and regression analysis [93,94,95]. The classifier that the SVM initializes is useful for predicting between two possible outcomes that depend on continuous or categorical predictor variable [96]. In 1995, Tin Kam Ho described the random forest algorithm, which is an ensemble learning method for classification, regression and other tasks, operated by constructing a large number of decision trees at training time and outputting the class that is the mode of the classes (classification) or mean prediction (regression) of the individual trees [97]. This method can correct for decision trees’ habit of overfitting to their training set [98].

### 3.2. AI Revolution Using Deep Learning in the Big Data Era

In the 21st century, we have access to large amounts of data, known as “Big Data”, and faster computer and advanced machine learning techniques were successfully applied to many problems throughout society, which accelerates social implementation of AI technologies. Indeed, by 2016, the market for AI-related products reached more than 8 billion dollars, and the New York Times reported that interest in AI had reached a “frenzy” [99]. In particular, advances in deep learning, a branch of machine learning that models high level abstractions in data by using a deep graph with many processing layers, drove progress and research in image and video processing, text analysis and even speech recognition (Figure 2) [100]. Artificial neural networks (ANNs) were inspired by information processing and distributed communication nodes in biological systems; meanwhile, ANNs have various differences from biological brains. Specifically, neural networks tend to be static and symbolic, while the biological brain of most living organisms is dynamic (plastic) and analog [101].

Importantly, the current progress of deep learning technologies has been truly astonishing. In 2012, AlexNet, which is the name of a convolutional neural network (CNN) designed by Alex Krizhevsky, won the ImageNet Large Scale Visual Recognition Challenge (ILSVRC); this network achieved a top-5 error of 15.3%, more than 10.8 percentage points lower than that of the runner up [102]. AlexNet achieved state-of-the-art recognition accuracy against all the traditional machine learning and computer vision approaches, which was a significant breakthrough in the field of machine learning and computer vision for visual recognition and classification tasks and the point in history where interests in deep learning rapidly increased [103]. With regard to the accuracy for ILSVRC, the error rate of the deep learning model designed by the winners’ group in each year has significantly been improved year by year. Particularly, ResNet-152, the 152-layer Residual Neural Network (ResNet), developed by Microsoft group achieved 3.57% error rate in 2015, which won the 1st place in the ILSVRC2015 and outperformed human accuracy (5% error rate) [103,104]. In addition to the superhuman performance of AlphaGo, an AI-powered system based on the deep reinforcement learning (DRL) technology that beat the world No.1 ranked Go player [105], now AI technologies using deep learning attract a lot of attention in the various kinds of fields, including the medical field [106,107,108].

## 4. Epigenetics Analysis and Integrated Analysis of Multiomics Data, Including Epigenetic Data, Using AI Technologies in the Medical Field

### 4.1. Advantages of Machine Learning and Deep Learning Technologies for Analysis of Medical Big Data

**Figure 3:**
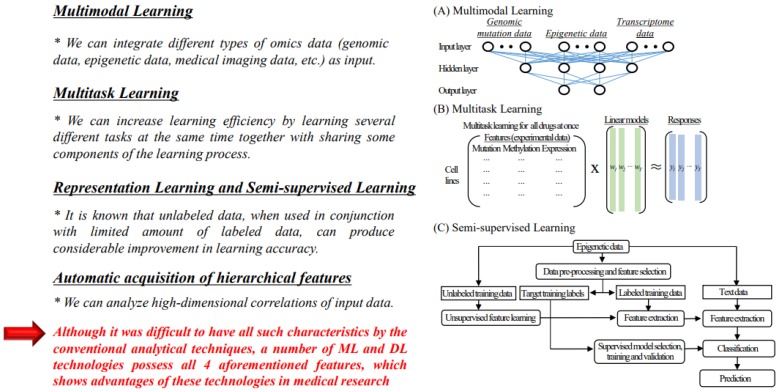
Advantages of machine learning and deep learning technologies in medical research. (**A**) An example of multimodal learning analysis using multiomics data including epigenetic data. (**B**) An example of multitask learning analysis using gene mutation data, DNA methylation data and gene expression data. This is a modified figure from reference [113]. (**C**) An example of semi-supervised learning using epigenetic data. This is a modified figure from reference [114].

In order to realize precision medicine, integrated analysis of medical big data is essential; we summarized the advantages of machine learning and deep learning technologies for analysis of medical big data (Figure 3). So far, it has been difficult to have all such characteristics by the conventional analytical techniques, but a number of machine learning and deep learning technologies possess all four features, which shows advantages of these technologies in medical research.

#### 4.1.1. Multimodal Learning

Data in the real world usually comes as different modalities. For instance, images are associated with captions and tags, videos contain visual and audio signals, sensory perception includes simultaneous inputs from visual, auditory, motor and haptic pathways [109]. Different modalities are characterized by very different statistical properties. For example, images are usually represented as pixel intensities or outputs of feature extractors, while texts are represented as discrete word count vectors. Given the distinct statistical properties of different information resources, to discover the relationship between different modalities is very important. Multimodal Learning is a good model to represent the joint representations of different modalities, such as genomic mutation data, epigenetic data and transcriptome data in medical research (Figure 3A). In fact, it was reported that predications of cancer prognosis or anti-cancer drug sensitivities were enabled based on multimodal learning using various different types of medical data [110,111,112]. Since molecular mechanisms of diseases like cancer are pretty complicated and a variety of factors are intricately involved, characteristics of multimodal learning must be critical for elucidation of the mechanism of diseases.

#### 4.1.2. Multitask Learning

Multitask Learning is a subfield of machine learning in which multiple learning tasks are solved at the same time, while exploiting commonalities and differences across tasks [115]. Using this approach, we can improve learning efficiency and prediction accuracy for the task-specific models, when compared to training the models separately [116,117]. Although multitask learning algorithms have a long history in machine learning, their common theme is that by sharing information between tasks, and often by encoding that the learned models for different tasks should have some similarity to each other [113,118]. It is possible to improve over independent training of individual tasks, in particular when training data for each task may be limited [113]. Intriguingly, several multitask learning approaches have recently been proposed to predict drug sensitivity; two kernel-based methods demonstrated improved performance over elastic net regression [113,119,120,121,122,123]. In this regard, a kernel-based multitask approach was the winner of a DREAM competition to predict drug sensitivity in a small breast cancer cell line data set [123], and another work encoded features of drugs in a neural network based multitask strategy [119]. For example, a schematic figure of multitask models is shown in Figure 3B (modified figure from reference [113]). In this case, trace norm regularization with a highly efficient ADMM (alternating direction method of multipliers) optimization algorithm that readily scales to large data sets was used. In the precision medicine era, because to predict drug sensitivity for each patient is a fundamental task, the concept of multitask learning to analyze omics data including epigenetic data is useful.

#### 4.1.3. Representation Learning and Semi-Supervised Learning

In machine learning, representation learning is a set of techniques that allows a system to automatically discover the representations needed for feature detection or classification from raw data [124]. This replaces manual feature engineering and allows a machine to both learn the features and use them to perform a specific task. Semi-supervised learning is a class of machine learning tasks and techniques that also make use of unlabeled data for training—typically a small amount of labeled data with a large amount of unlabeled data [125]. On the basis of these characteristics, it is known that unlabeled data, when used in conjunction with limited amount of labeled data, can produce considerable improvement in learning accuracy [126,127]. A flowchart of the training and testing processes of a semi-supervised deep learning method for cancer prediction is shown in Figure 3C (modified figure from reference [114]). The semi-supervised classification model consists of the unsupervised feature extraction stage and the supervised classification stage, which is possible to address both unlabeled and labeled data to extract more valuable information and make better predictions [114]. As the number of labeled data is often limited in the medical data, particularly for analysis of rare diseases, this characteristic is useful in such case.

#### 4.1.4. Automatic Acquisition of Hierarchical Characteristics

Deep learning is a type of machine leaning technique that aims at learning feature hierarchies with features from higher levels of the hierarchy formed by the composition of lower level features. Automatically learning features at multiple levels of abstraction enable construction of a system to learn complex functions mapping the input to the output directly from data, without relying completely on human-crafted features [128].

### 4.2. Analysis of Epigenetic Data and Integrated Analysis of Epigenetic Data and Other Omics Data Using AI Technologies

Although the screening of genetic mutations is considered common practice for testing an individual’s predisposition to cancer, it cannot reflect the current status or activity of disease [129,130]. In contrast, promoter DNA methylation is a more systematic method for evaluation due to its defined location within the promoter regions of specific genes. In general, locating gene mutations is more complex as they can occur at unsuspected sites within the gene that may be challenging to pinpoint. In this regard, several epigenetic markers have value in the early detection of cancers based on their involvement in the initiation of carcinogenic pathways [129,131,132]. Hence, epigenetic biomarkers are likely to have great potential and wide scope to be implemented as diagnostic biomarkers. Consequently, we can expect that the strategy of combining epigenetic biomarkers and AI technologies (machine learning and deep learning technologies) might be useful for the diagnosis of diseases.

Brain tumors are clinically and biologically highly diverse, which encompasses a wide spectrum from benign tumors, which can frequently be cured by surgery alone (e.g., pilocytic astrocytoma), to highly malignant tumors that respond poorly to any therapy (e.g., glioblastoma) [133,134]. So far, a number of studies reported substantial inter-observer variability in the histopathological diagnosis of a lot of central nervous system (CNS) tumors, for instance, in diffuse gliomas, ependymomas and supratentorial primitive neuroectodermal tumors [133,135,136,137]. Since DNA methylation profiling is robust and reproducible even from small samples and poor quality material [138], DNA methylation profiles have been widely used to subclassify CNS tumors that were previously considered homogenous diseases [133,137,139,140,141,142,143,144]. On the basis of this previous work within single entities, Capper et al. recently presented a comprehensive machine learning approach for the DNA methylation-based classification of central nervous system tumors across all entities and age groups, and demonstrated its application in a routine diagnostic setting [133]. This study showed that the availability of the DNA methylation-based classification method using machine learning might have a substantial impact on diagnostic precision compared to standard methods, which results in a change of diagnosis in up to 12% of prospective cases [133]. In essence, this study provides new strategy for the generation of epigenetics-based tumor classifiers using AI across other cancer entities, with the potential to fundamentally transform tumor pathology.

Integrated analysis of epigenetic data with other omics data using AI technologies has also been advanced [110,111,112]. For example, Chaudhary et al. presented a deep learning-based model on hepatocellular carcinoma (HCC) robustly differentiated survival subpopulations of patients in six cohorts; they built the deep learning-based survival-sensitive model on 360 HCC patients’ data using epigenetics (DNA methylation) data with RNA sequencing (RNA-seq) data and microRNA-sequencing (miRNA-seq) data from The Cancer Genome Atlas (TCGA), which predicts prognosis as good as an alternative model where genomics and clinical data are both considered [111]. In this case, the autoencoder method, which is an unsupervised deep learning technique, was used in the model, and it could capture sufficient variations due to potential clinical risk factors, such that it performs as accurately or even better than, having additional clinical features in the model 111]. Importantly, the autoencoder framework showed much more efficiency to identify features linked to survival compared with the principal component analysis (PCA) or individual Cox proportional-hazards-based models [111].

A fundamental integrated analysis of epigenetic data with other omics data using AI technologies is that we can clarify the significance of genetic mutations in the noncoding regions of the human genome. Although genome-wide association studies (GWAS) have already identified a large number of inherited risk loci for cancer susceptibility, many of these single-nucleotide polymorphisms (SNPs) reside in a noncoding genome within known DNA regulatory elements [82]. However, the majority of annotation tools only annotate SNPs in the coding region of a genome [145,146]. This is in part because noncoding SNPs are more challenging to annotate than SNPs in coding regions where the consequences of variation are better understood [145]. In order to predict functional SNPs in a noncoding genome, Corces et al. recently presented the genome-wide chromatin accessibility of 410 tumor samples spanning 23 cancer types from TCGA; they identified 562,709 transposase-accessible DNA elements that substantially extend the compendium of known cis-regulatory elements [82]. The integrated analysis of ATAC-seq with TCGA data identified numerous putative distal enhancers that can distinguish molecular subtypes of tumors, uncovered specific driving transcription factors through protein-DNA footprints, and nominated long-range gene-regulatory interactions in tumors [82]. The findings by group of Corces and others reveal genetic risk loci of cancer predisposition as active DNA regulatory elements in cancer can identify gene-regulatory interactions underlying cancer immune evasion and pinpoint noncoding mutations that drive enhance activation and may affect patient survival. These results suggest a systematic approach to understanding the noncoding genome in cancer to advance diagnosis and therapy. In their study, K-means clustering was used, being one of the simplest and most popular unsupervised machine learning algorithms [82]. Meanwhile, given that whole genome sequencing (WGS) analysis using a large number of cancer tissues is being actively conducted worldwide, the development of AI-based platforms that can perform integrated analyses of large-scale multiomics data must be critical to finding useful information for the diagnosis and therapy of cancer.

As mentioned above, the Hi-C technique emerged as a powerful tool for studying the spatial organization of chromosomes, as it measures all pair-wise interaction frequencies across the entire genome [127]. During recent years, this method facilitated a number of significant discoveries like A/B compartment, topological associating domains (TADs), chromatin loops and frequently interacting regions (FIREs), which significantly expanded understandings of three-dimensional (3D) gene organization and gene regulation machinery [61,147,148,149,150,151]. However, the Hi-C technology usually requires an extremely deep-sequencing depth to achieve high resolution; this fact causes the remarkable rise of experimental costs, which makes it hard for researchers to apply it to a large number of cell lines [149,152,153]. In this regard, several computational methods have been reported to improve the resolution of Hi-C data and detect physiological interactions at the regulatory element level [152,154,155,156,157]. For example, Zhu et al. reported EpiTensor, which is a high-order tensor decomposition based algorithm to identify 3D spatial associations within TADs from 1D maps of histone modifications, chromatin accessibility and RNA-seq [155]; Whalen et al. presented a machine learning pipeline called TargetFinder, which integrates data for annotation, Cap Analysis of Gene Expression (CAGE), ChIP-seq, DNase I hypersensitive sites sequencing (DNase-seq), FAIRE-seq (Formaldehyde-Assisted Isolation of Regulatory Elements) and DNA methylation to predict individual promoter-enhancer interactions across the genome [157]. Additionally, Bkhetan et al. introduced a supervised learning pipeline called 3DEpiLoop, which uses random forest as a statistical learning algorithm, and this algorithm can predict three-dimensional chromatin looping interactions within TADs from one-dimensional epigenetics and transcription factor profiles using the statistical learning [156]. Zhang et al. also developed HiCPlus, which is a computational approach based on the deep convolutional neural network, to infer high-resolution Hi-C interaction matrices from low-resolution Hi-C data [154]. More recently, Li et al. developed a bootstrapping deep learning model called DeepTACT (deep neural networks for chromatin conTACTs predictions), which can predict chromatin contacts at individual regulatory element level using sequence features and chromatin accessibility information [152]. This model employed a bootstrapping strategy, which is based on the theory established in the paper reported by Wallace et al. in 2011 [158]. In essence, DeepTACT can predict not only promoter–enhancer interactions, but also promoter–promoter interactions, and DeepTACT fine-maps chromatin contacts of high-quality promoter capture Hi-C (PCHi-C) from the multiple regulatory element level (5–20 kb) to the individual regulatory element level [130]. In addition, DeepTACT can identify a set of hub promoters, which are active across cell lines, enriched in housekeeping genes, closely related to fundamental biological processes and capable of reflecting cell similarity [152]. The other important advantage of this model is that we can infer novel associations for coronary artery disease through integrative analysis of chromatin contacts predicted by DeepTACT and existing GWAS, which provides a powerful way to build a fine-scale chromatin connectivity map to explore the mechanisms of human diseases [152]. As noted above, because most of the non-coding variants are not well annotated linked to genes that they regulate, it is still difficult to evaluate the significance of these mutations. Hence, precise identification of interactions between promoters and their regulation is urgently needed; aforementioned integrated analysis of DeepTACT-based chromatin contacts and GWAS-based gene mutation data appears to be pretty important.

### 4.3. Issues of AI Technologies for Omics Analysis

Thus far we introduced a number of merits to use AI technologies for integrated analysis of omics data; but there are also several defects we need to overcome in them. One of the serious issues of AI technologies is a phenomenon called overfitting. In general, overfitting means that the production of an analysis that corresponds too closely or exactly to a particular set of data, which sometimes causes the failure of fitting additional data or predicting future observations reliably [159]. Overfitting in neural networks shows poor performance on the test set compared to the training data set, signifying a loss of generalization. More specifically, the model learns the noise patterns present in the training data set, thereby causing a large gap between the training and test error [160]. Principally, deep neural networks are prone to overfitting because of the large number of parameters to be learned [160]; additionally, these networks are so flexible and overparameterized that they adjust the parameters in order to fit the training data even with labels randomized [160,161,162].

Meanwhile, in order to avoid overfitting, several methods have also been proposed, and we highlight some important methods below:

Cross-validation: Cross-validation is any of various similar model validation techniques for assessing how the results of a statistical analysis will generalize to an independent data set. It is used to test the effectiveness of a machine learning model and is also a resampling procedure used to evaluate a model if we have a limited data. It was reported that cross-validation could reduce the risk of selecting models that suffer from overfitting to the observed data [163].

Regularization: An appropriate level of complexity is needed to avoid overfitting, and regularization is a method that controls a model’s complexity by penalizing the magnitude of its parameters [164]. The common regularization methods to reduce overfitting are L1 regulation (a regression model that uses L1 regulation technique is called Lasso Regression), L2 regulation (a regression model that uses L2 regulation technique is called ridge regression), dropout regulation (reducing overfitting in neural networks by preventing complex co-adaptations on training data) and early stopping (stopping the model when model reaches a plateau) [165].

Train with more data: Even though it is not always available, training with more data can help algorithms detect the signal better. In the earlier example of modeling height vs. age in children, it is clear how sampling more schools can help our model. However, an important point we should be careful about is that this is not always the case because this method cannot help our model if we just add noisy data. Therefore, that is why we should always ensure our data are clean and relevant.

The other critical issue of using AI technologies for omics analysis is that omics data including genomic data and epigenetic data possess a large number of parameters (for example, the number of human genes are around 30,000), which are often much higher than that of sample number. Especially, in the case of rare diseases, the number of patients is critically small; but current aforementioned WGS technology enables researchers to interrogate all three billion base pairs of the human genome. This kind of problem is generally typified as the “curse of dimensionality”; the number of features characterizing the data are “too large” and “the curse of dataset sparsity”; the number of samples on which these features are measured is “too small” [166]. The curse of dataset sparsity refers to the scenario where the number of parameters like genomic factors is far larger than the number of samples, which results in model overfitting and computational inefficiency [167]. In order to overcome this “curse of dimensionality” issue for omics analysis, new techniques have also been developed. Recently, regularized logistic regression using the L1 regularization has successfully applied in high-dimensional cancer classification to tackle both the estimation of gene coefficients and the simultaneous performance of gene selection; but the L1 regularization has a biased gene selection and does not have the order property. Hence, Wu et al. investigated the L1/2 regularized logistic regression for gene selection in cancer classification, and experimental results on three DNA microarray database demonstrated the proposed method using sparse logistic regression with L1/2 regularization outperformed other commonly used sparse methods (L1 regulation and elastic net penalty) in terms of classification performance [168]. Furthermore, Romero et al. developed a novel deep learning-based technique called diet networks, which could considerably reduce the number of free parameters [169]. This model is based on the idea that we can first learn or provide a distributed representation for each input feature (e.g., for each position in the genome where variations are observed in data), and then learn (with another neural network called the parameter prediction network) how to map a feature’s distributed representation (on the basis of the feature’s identity) to the vector of parameters specific to that feature in the classifier neural network (the weights which link the value of the feature to each of the hidden units) [169], which could deal with the issues of producing the parameters associated with each feature as a multitask learning model [169]. Given that the diet networks algorithm enables significant reduction of both the number of parameters and the error rate of the classifier, it must be useful to apply this method for analysis of various type of omics data, including epigenetic data.

## 5. Concluding Remarks and Future Perspectives

In this review, we discussed the current conditions and possibilities of omics analyses using AI for the realization of precision medicine. In particular, we focused on epigenetics analysis. As mentioned, omics analyses using AI technology have many possibilities; however, there are a number of issues we need to overcome. In this regard, we thought that AI-based techniques need to be improved for their successful application to realization of precision medicine, based on the efforts to solve current issues one by one. One important strategy seems to be that experts of biomedical science, and experts of information science and bioinformatics should collaborate deeply. In this way, both groups of experts can solve problems together based on highly merged knowledge because, definitely, this is an interdisciplinary research field. In addition, while the progress of AI algorithms is important, we thought that it is also critical to construct a database for the accumulation of a large quantity of omics data, where high-quality appropriate annotation is added with right clinical information. After all, even if a huge number of omics data is available, poor quality of big data would create misleading results. As it is thought that the practical use of AI is indispensable to the realization of precision medicine, we believe that continuing efforts to solve the issues we have mentioned herein surely and steadily will contribute to the realization of true precision medicine.

## Figures and Tables

**Table 1:** List of main technologies for epigenetics and chromatin analyses.

Method Name	Purpose	Methodology	Era	Ref.
Chromatin immunoprecipitation (ChIP) assay	Analysis of histone modification and transcription factor binding status	A type of immunoprecipitation experimental technique used to investigate the interaction between proteins and DNA in the cell. It aims to determine whether specific proteins are associated with specific genomic regions, and also aims to determine the specific location in the genome that various histone modifications are associated with.	1985	[39,40]
Bisulfite sequencing (BS-Seq)	DNA methylation analysis	Treatment of DNA with bisulfite converts cytosine residues to uracil, but leaves 5-methylcytosine residues unaffected. Hence, DNA that has been treated with bisulfite retains only methylated cytosines.	1992	[41,42]
Histone acetyltransferase (HAT) assay	Assay for histone acetyltransferase activity	Multiple biochemical HAT assays have been described; these assays measure HAT activity by detecting either the acetylated histone-based product (direct) or the free CoA product (indirect).	1995	[43,44]
DNA methylation array: differential methylation hybridization (DMH)	DNA methylation analysis	A DNA array-based method, called differential methylation hybridization (DMH), to identify hypermethylated sequences in tumor cells by simultaneously screening many CpG island loci derived from a genomic library, CGI.	1999	[45,46]
ChIP-on-chip	Genome-wide analysis of histone modification and transcription factor binding status	A technology that combines chromatin immunoprecipitation (ChIP) with DNA microarray (chip). It allows the identification of the cistrome, the sum of binding sites, for DNA-binding proteins on a genome-wide basis.	1999	[47,48]
Histone methyltransferase (HMT) assay	Assay for histone methyltransferase activity	Radiometric Assays, Mass Spectrometry, Anti-Methylation Antibody-Based Detection, Enzyme-Coupled SAH Detection, Protease-Coupled Detection, Competition Binding.	2000	[49,50]
Histone demethylase (HDMT) assay	Assay for histone demethylase activity	Measuring the release of radiolabeled formaldehyde from ^3^H-labeled methylated histone substrates, by monitoring the change in methylation levels of histone substrates by immunoblotting with site-specific methyl-histone antibodies, or by using mass spectrometry to detect reductions in histone peptide masses that correspond to methyl groups.	2004	[51,52]
Reduced Representation Bisulfite Sequencing (RRBS)	Genome-wide DNA methylation analysis	An efficient and high-throughput technique for analyzing the genome-wide methylation profiles on a single nucleotide level; it combines restriction enzymes and bisulfite sequencing to enrich for areas of the genome with a high CpG content.	2005	[53,54]
ChIP-loop	Chromosome conformation capture technique	This method combines the standard 3C protocol with a routine ChIP protocol; it allows the selective identification of long-range chromatin interactions between loci that are bound to specific proteins of interest.	2005	[55,56]
ChIP-sequencing (ChIP-seq)	Genome-wide analysis of histone modification and transcription factor binding status	By combining chromatin immunoprecipitation (ChIP) assays with next-generation sequencing (NGS), ChIP sequencing (ChIP-seq) is a powerful method for identifying genome-wide DNA binding sites for transcription factors and other proteins.	2007	[57,58]
Whole Genome Bisulfite Sequencing (WGBS)	Genome-wide DNA methylation analysis	A NGS technology used to determine the DNA methylation status of single cytosines by treating the DNA with sodium bisulfite before sequencing.	2009	[59,60]
Hi-C	Chromosome conformation capture technique	A genome-wide chromatin conformation capture protocol using proximity ligation. The technology is of special interest for three-dimensional genome organization in the nucleus and de novo genome assemblies.	2009	[61,62]
ChIA-PET	Determination of de novo long-range chromatin interactions genome-wide	The ChIA-PET method combines ChIP-based methods, and Chromosome conformation capture (3C), to extend the capabilities of both approaches.	2009	[63,64]
ATAC-seq	Identification of accessible DNA regions	This method relies on NGS library construction using the hyperactive transposase Tn5. NGS adapters are loaded onto the transposase, which allows simultaneous fragmentation of chromatin and integration of those adapters into open chromatin regions.	2013	[65,66,67,68,69,70,71,72,73,74]
Capture Hi-C (CHi-C)	Identification of higher resolution mapping of chromatin interactions	The CHi-C is a new technique for assessing genome organization based on chromosome conformation capture coupled to oligonucleotide capture of regions of interest like gene promoters.	2014	[75]
